# Improved Bevirimat resistance prediction by combination of structural and sequence-based classifiers

**DOI:** 10.1186/1756-0381-4-26

**Published:** 2011-11-14

**Authors:** J Nikolaj Dybowski, Mona Riemenschneider, Sascha Hauke, Martin Pyka, Jens Verheyen, Daniel Hoffmann, Dominik Heider

**Affiliations:** 1 Department of Bioinformatics, Center of Medical Biotechnology, University of Duisburg-Essen, Universitaetsstr. 2, 45117 Essen, Germany; 2 Leibniz Institute for Arteriosclerosis Research, University of Münster, Domagkstr. 3, 48149 Münster, Germany; 3 CASED, Technische Universität Darmstadt, Mornewegstr. 32, 64293 Darmstadt, Germany; 4 Department of Psychiatry, University of Marburg, Rudolf-Bultmann-Str. 8, 35039 Marburg, Germany; 5 Institute of Virology, University of Cologne, Fuerst-Pueckler-Str. 56, 50935 Cologne, Germany

## Abstract

**Background:**

Maturation inhibitors such as Bevirimat are a new class of antiretroviral drugs that hamper the cleavage of HIV-1 proteins into their functional active forms. They bind to these preproteins and inhibit their cleavage by the HIV-1 protease, resulting in non-functional virus particles. Nevertheless, there exist mutations in this region leading to resistance against Bevirimat. Highly specific and accurate tools to predict resistance to maturation inhibitors can help to identify patients, who might benefit from the usage of these new drugs.

**Results:**

We tested several methods to improve Bevirimat resistance prediction in HIV-1. It turned out that combining structural and sequence-based information in classifier ensembles led to accurate and reliable predictions. Moreover, we were able to identify the most crucial regions for Bevirimat resistance computationally, which are in line with experimental results from other studies.

**Conclusions:**

Our analysis demonstrated the use of machine learning techniques to predict HIV-1 resistance against maturation inhibitors such as Bevirimat. New maturation inhibitors are already under development and might enlarge the arsenal of antiretroviral drugs in the future. Thus, accurate prediction tools are very useful to enable a personalized therapy.

## Background

### HIV and Bevirimat

Bevirimat (BVM) belongs to a new class of antiretroviral drugs inhibiting the maturation of HIV-1 particles to infectious virions. BVM prevents the final cleavage of precursor protein p25 to p24 and p2. In electron microscopy, these immature particles failed to build a capsid composed of a nucleocapsid (p7) and RNA surrounded by a cone-shaped core assembled from p24 proteins [1]. In selection experiments with BVM mutations at Gag cleavage site p24/p2 BVM resistance emerged and was conferred in phenotypic resistance tests. In contrast, especially natural polymorphisms in the QVT-motif of p2 hampered the effective suppression of viral replication in clinical phase II trails, which also increased the measured resistance factors in cell culture experiments. It was recently shown by Keller *et al. *that BVM stabilizes the immature Gag lattice and thus, prevents cleavage [2].

Machine learning techniques are widely used to predict drug resistance in HIV-1. For instance, Beerenwinkel *et al. *used support vector machines [3] and decision trees [4] to predict drug resistance of HIV-1 against several protease and reverse transcriptase inhibitors. Other groups also employed artificial neural networks [5,6], rule-based systems [7] and random forests [8,9].

In our recent publication, we demonstrated the use of machine learning techniques for the prediction of Bevirimat resistance from genotype [10]. We tested artificial neural networks, support vector machines, rule-based systems and random forests [11] trained on p2 sequences derived from resistant and susceptible virus strains and applied different descriptor sets. Descriptors map the amino acid symbols onto numerical values representing physico-chemical properties of the amino acids. Due to the fact that the p2 sequences have insertions and deletions and thus differ in their length, they were preprocessed to fulfill the constraints given by machine learning approaches, i.e. a fixed input dimension of the data. We used a multiple sequence alignment to align and subsequently encode the sequences with five different descriptors, namely the hydrophobicity scale of Kyte and Doolittle [12], molecular weight, isoelectric point, pKa and HIV-1 cleavage probability [13]. Finally, all models were trained using the encoded protein sequences and evaluated using 100-fold leave-one-out cross-validation. The random forest models trained on hydrophobicity-encoded p2 sequences performed best (AUC = 0.927 ± 0.001) with regard to Wilcoxon signed-rank tests on the AUC distributions. Moreover, earlier studies [14] have shown that RFs are highly stable and robust in comparison with other classifiers. RFs also provide an importance estimation for the variables in the data set. The importance of each variable, i.e. sequence position in p2, can be assessed to gain a possible biological implications on resistance mechanisms.

### Classifier Ensembles

Classifier ensembles have been shown to often lead to better prediction performance compared to single classifiers in several studies [15,16]. The random forests models used in our initial study are already examples of classifier ensembles, consisting of independent decision trees that are based on the same feature set. However, classifier ensembles can also be constructed by combining different datasets or different representations (here descriptors) of the same data. In order to combine the outputs of single classifiers for a final decision of an ensemble, several fusion methods have been proposed, ranging from simple mathematical functions, such as min and max, to second-level learning [9], also called stacking [17]. In the quest for optimal classifier ensembles, genetic algorithms (GA) have been suggested in various studies [18-20]. Genetic algorithms mimic the idea of evolution and its natural processes of mutation, recombination and selection of individuals. GAs are used to heuristically solve optimization problems with a complex fitness landscape and are frequently applied in biomedical research [21-23]. The central components of a genetic algorithm are the population and the fitness function evaluating the individuals (chromosomes) therein. During each generation the best, i.e. most fit, individuals (*parents*) are selected by methods such as stochastic universal sampling or tournament selection to generate a new generation of slightly varied *offspring*. Variations are introduced through so-called genetic operators, e.g. mutation or recombination, that impose the genetic variability and sample the fitness landscape. Generations of individuals are established until one or more termination conditions are reached.

## Material and methods

### Data

In this study we used the data aggregated by Heider *et al. *[10], consisting of p2 sequences of viruses with assay-determined resistance factors. These data were collected from several studies that have investigated polymorphisms in the p2 region by phenotypic BVM resistance assays. The cut-off value of the resistance factor used to define the classes "resistant to BVM" and "susceptible to BVM" was set according to Heider *et al. *[10]. Duplicate sequences in each of the classes were removed prior to analysis. The final dataset consisted of 43 p2 sequences of HIV-1 strains with susceptibility or intermediate resistance to BVM and 112 sequences of resistant strains. The lengths of the p2 sequences in the data set are 20.77 ± 0.43. The p2 sequences have a very low sequence identity/similarity. Only six positions within the peptides are conserved, namely 359-361 and 365-367. Position 357 shows only small similarity, whereas position 358 and 362 show higher similarity among the sequences. All other positions, especially in the N-terminal part, are highly diverse. The highly conserved regions are marked with an asterisk in the wildtype sequence:

GHKARVLAEAMSQVTNSATIM

.:***: ***

### Machine Learning

#### Performance evaluation

All models used in this study were evaluated with 10-fold leave-one-out cross-validation. As performance measurement we used the area under the Receiver Operating Characteristics curve (AUC) [24], i.e. the integral under the ROC curves. ROC curves are built by calculating sensitivity and specificity for every possible cutoff between the positive (here susceptible) and negative (here resistant) samples. ROC curves were drawn with R-package ROCR [25]. Table 1 gives an overview of the areas under the curves (AUCs). After counting true positives *TP*, false positives *FP*, false negatives *FN *and true negatives *TN*, sensitivity, specificity and accuracy are calculated according to the standard definitions reported below for the sake of completeness:

**Table 1:** Area under the curve

method	mean AUC	95% CI
RF [10]	0.927	0.002

RF.293	0.944	0.003

RF.ESP	0.898	0.006

GAD	0.947	0.004

CE1.max	0.946	0.004
CE1.min	0.943	0.006
CE1.product	0.947	0.004
CE1.mean	0.947	0.004

CE2.max	0.956	0.004
CE2.min	0.954	0.004
CE2.product	0.956	0.004
CE2.mean	0.955	0.004
CE2.stacking	0.933	0.005

RF.ESP+293.max	0.946	0.001
RF.ESP+293.min	0.945	0.001
RF.ESP+293.product	0.956	0.001
RF.ESP+293.mean	0.958	0.001
RF.ESP+293.stacking	0.930	0.006

CE.GA	0.954	0.006
CE.GA.RF	0.949	0.005

Results of the 10-fold leave-one-out cross validation. 95% CI: 95% confidence interval.

(1) $$sensitivity=\frac{TP}{TP+FN}$$

(2) $$specificity=\frac{TN}{TN+FP}$$

(3) $$accuracy=\frac{TP+TN}{TP+TN+FP+FN}$$

Models were compared based on their resulting AUC distributions from the 10-fold leave-one-out cross-validation runs using Wilcoxon signed-rank tests [26,27]. The null hypothesis was that there are no differences between the compared classifiers. 95% confidence intervals of the AUC were calculated by *t*-testing.

#### Descriptor Encoding

The Interpol package [28] was used to encode p2 sequences using all 531 descriptor sets of the AAindex database [29] and to normalize the feature length using a linear interpolation [30], resulting in 531 numerically encoded datasets. The feature length was set to 21, representing the most common sequence length found in the dataset.

#### Evolutionary Optimization of Descriptors

Individuals were represented as vectors of twenty numerical values (*genes*), encoding the proteinogenic amino acids. The mutation probability for each gene was 0.01. Recombination was applied with a probability of 0.1. Recombination partners were chosen according to the fitness proportionate selection operator. The fitness of an individual was given by its BVM resistance classification performance (AUC). The GA was run thrice for 1000 generations.

#### Random Forests

The randomForest package [31] of R [32] was used to build all RF models used in this study. Each RF consisted of 500 decision trees that were combined by majority voting. Feature importance was assessed using the built-in function of the randomForest package and estimated by the *sum of all decreases in Gini impurity*, which has been shown to be more robust compared to the *mean decrease in accuracy *[33].

#### Classifier Ensembles

Classifier ensembles CE1 and CE2 were constructed from the set of 531 single classifiers based on the different descriptors in the AAindex database. All single classifiers with an AUC of > 0.93 and > 0.94 were included into CE1 and CE2, respectively. The votes of the single classifiers within CE1 and CE2 were combined by applying simple methods such as min, max, product and mean to reach a final decision. In addition, the classifier ensembles were stacked using a random forest model trained on the outcomes of the single classifiers.

The evolutionary optimization of classifier ensembles was similar to that of a descriptor set, described earlier. The fitness of an ensemble was represented by the resulting performance of that ensemble on the BVM resistance prediction (represented by the AUC). An individual consisted of a set of unique classifiers. Possible classifiers included artificial neural networks, support vector machines [34], k-nearest neighbors, decision trees and random forests. The minimum and maximum number of classifiers within an ensemble was set to 2 and 10, respectively. Mutations, e.g. insertions or deletions of classifiers, as well as changes to parameters in the specific machine-learning methods, were set to occur at a rate of 0.2. Each of the 100 generations comprised 15 individuals. The resulting classifier ensemble was termed CE.GA. In addition, a second ensemble including only RF models was created applying the same parameters and termed CE.GA.RF.

#### Structural classification

Homology models of all p2 sequences were built based on the NMR structure of the p2 α-helix [35] using Modeller 9.8 [36]. The electrostatic hull, representing the discretized electrostatic potential *φ*(*r*) above the solvent-accessible surface was calculated as described in the original publications [9]. The resulting hull, calculated for each p2 model consisted of 200 *φ*(*r*)-values at a distance of approximately 0.6 nm above the solvent-accessible surface. Electrostatic potential vectors of the form (*φ*(*r*_1_),..., *φ*(*r*_200_)) were then used to train initial RF models. To cope with the unfavorable ratio of samples (n = 155) and features (p = 200) [37], a feature selection scheme was applied. The most important features, i.e. *φ*(*r*), as estimated by the RF internal importance analysis [11], were averaged over ten RF models and sorted in descending order. In an iterative manner, RF models were then built using feature subsets, starting with the most important and adding one additional feature per round. In each round the AUC was calculated.

## Results and Discussion

**Figure 1:**
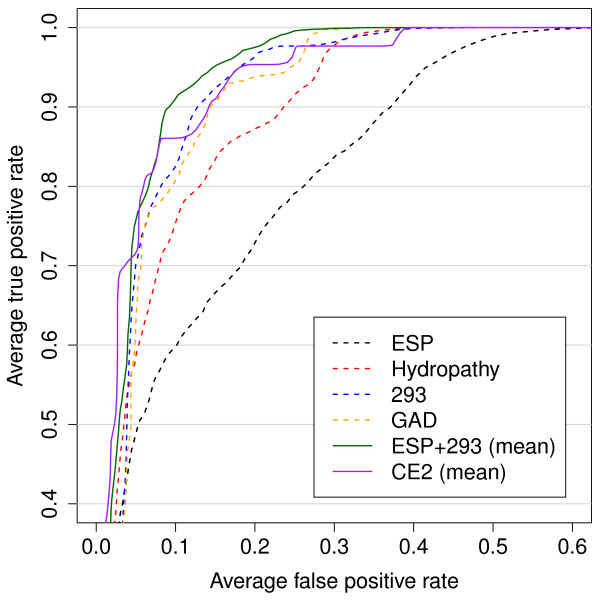
**ROC curves**. Performance comparison of single descriptor classifiers (dashed lines) and fused classifiers (solid lines). The hydropathy scale classifier was used in the original study [10]. The combined classifiers CE2 and ESP+293 achieve an AUC higher than any of the single descriptor classifiers (compare Table 1). Despite the inferiority of ESP as a single classifier, fusion with 293 results in the best overall performance.

The prediction qualities in form of the mean AUCs and 95% confidence intervals (CI) are shown in Table 1. ROC curves are shown in Figure 1. The best single classifier RF.293 (random forest trained with descriptor 293 [38]) reached an *AUC = *0.944 ± 0.003 for the prediction of Bevirimat resistance from p2 sequences and thus outperformed our recently published model that uses the hydrophobicity scale of Kyte and Doolittle [12] as descriptor *(AUC = *0.927 ± 0.001) with regard to the Wilcoxon signed-rank test. The best descriptor resulting from the genetic algorithm optimization reaches a fitness (i.e. AUC value) of 0.947 ± 0.002 and, thus, is only slightly better than the best descriptor 293. The optimized descriptor values representing the proteinogenic aminoacids are shown in Table 2. We then calculated the pairwise correlation of the novel descriptor GAD (genetic algorithm descriptor) with all other descriptors in the AAindex database [29]. It turned out that the highest correlation *(R*^*2 *^= 0.384) could be found with descriptor 452, i.e. the hydropathy scale of Naderi-Manesh *et al. *[39]. However, the RF trained on this hydropathy scale reached only an AUC of 0.928 ± 0.003, which is comparable to results obtained from the RF trained on the hydropathy scale of Kyte and Doolittle [12] in our recent publication [10]. Surprisingly, the GAD descriptor grouped the amino acids into pairs, except for alanine, cysteine, aspartic acid and serine. These groups represent a certain kind of similarity in the context of the novel descriptor.

**Table 2:** GAD values

amino acid	value	amino acid	value
A	0.0956	L	0.8577
R	0.8571	K	0.2695
N	0.9697	M	0.6212
D	0.1930	F	0.7062
C	0.2472	P	0.7154
Q	0.7865	S	0.5001
E	0.5843	T	0.2675
G	0.8036	W	0.4322
H	0.9811	Y	0.3246
I	0.3265	V	0.4513

The genetic algorithm optimized descriptor values for the 20 amino acids.

After the classification with single classifiers, we tested classifier ensembles for the prediction of BVM resistance. The classifier ensemble CE1 was built based on those single RFs reaching an average *AUC *> 0.93, resulting in 112 classifiers. The ensemble CE2 was built on a subset of CE1, namely those classifiers reaching an average *AUC *> 0.94 (11 classifiers). We then analyzed the correlation of the best classifier (293) with the other 10 classifiers in CE2 in two different ways. First, we calculated the correlation (cor.res) based on the votes for each protein sequence in the dataset, and second the correlation based on the 20 descriptor values (cor.des) for the 20 amino acids. The results are shown in Table 3. CE1 reaches AUC values of 0.946 ± 0.002, 0.943 ± 0.003, 0.947 ± 0.002 and 0.947 ± 0.002 for the fusion methods max, min product and mean, respectively. Obviously, a combination of these 112 RFs did not improve the prediction performance compared to RF.293. In contrast, CE2 outperformed CE1, as well as RF.293. The AUC values are 0.956 ± 0.002, 0.954 ± 0.002, 0.956 ± 0.002 and 0.955 ± 0.002 for max, min product and mean, respectively. Stacking of single classifiers of CE2 leads to a significant drop in prediction performance (*AUC *= 0.933 ± 0.002). This phenomenon has already been described by Dzeroski and Zenko [40], who demonstrated empirically on several machine learning methods and thirty datasets that stacking performs only comparable or even worse than picking the best single classifier. Nevertheless, stacking is controversely discussed as it sometimes lead to a better prediction performance. Ting and Witten [41] suggested the use of linear classifiers as fusion method for better generalization. This is in line with other findings that fusion with a non-linear random forest is inferior to the linear fusion with mean. The classifier ensembles built on the genetic algorithm procedure, CE.GA and CE.GA.RF reached AUC values of 0.954 ± 0.003 and 0.949 ± 0.002, respectively. Surprisingly, the homogeneous CE.GA.RF performs worse compared to CE2. We assume that the genetic algorithm was probably caught in a local maximum in the energy landscape, and thus, produced a suboptimal ensemble.

**Table 3:** Correlation analyses

descriptor	**cor.res (*R***^**2**^**)**	**cor.des (*R***^**2**^**)**
42	0.835	0.497
124	0.945	0.122
134	0.893	0.010
136	0.891	0.018
137	0.883	0.019
164	0.833	0.085
225	0.817	0.068
293	1.000	1.000
368	0.855	0.010
424	0.863	0.500
478	0.822	0.009

Correlation between the best descriptor (293) and the other 10 descriptors in CE2 were calculated. cor.res: correlation R based on the votes for each protein sequence in the dataset; cor.des: correlation R based on the descriptor values for the 20 amino acids.

**Figure 2:**
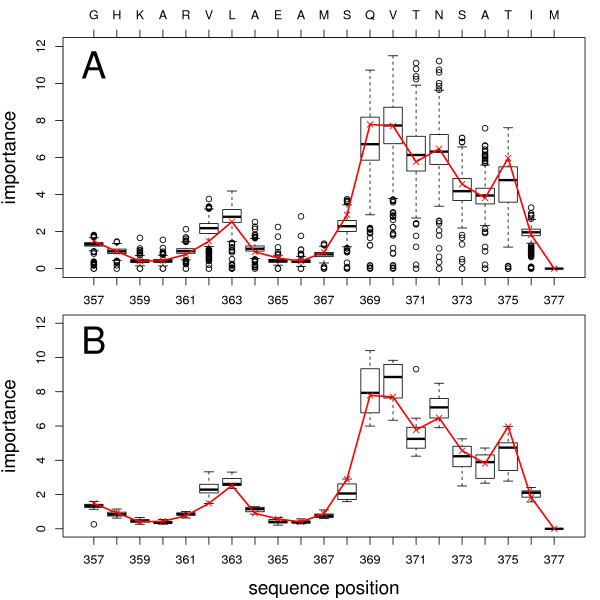
**Importance of sequence positions**. Importance of sequence positions in p2 for prediction of Bevirimat resistance. The y-axis denotes the "sum of all decreases in Gini impurity" [11]. The upper horizontal axis indicates wild type sequence. A: importance analysis over all descriptors; B: importance analysis of CE2 descriptors; The red lines mark the importance analysis for RF.293.

The most important features for the successful classification of the single RFs highlight sequence positions in the C-terminal end of the p2 sequence, specifically at sequence positions 369-376 (Figure 2). These findings are in agreement with our recently published results [10]. In the wild type sequence this region corresponds to the motif QVTNSATI. However, the analysis of the complete set of descriptors showed that the importance of positions 369-372 is in strong agreement with the findings of van Baelen *et al. *who identified the QVT motif at positions 369-371 as important [42]. In contrast, results obtained with the hydrophobicity scale as descriptor, are only in partial agreement with the experimental results [10]. Thus, we recommend to analyze the importance measurement for all available descriptors simultaneously to get reliable estimations. Positions 363 and 364, although being identified as crucial [43] for the resistance to BVM, only showed a slightly higher importance compared to the surrounding positions. This could be due to the nature of our dataset as already discussed in our recent study [10].

In order to test the predictive performance of a structural classifier, we calculated the electrostatic potential resulting from p2 sequences as proposed by Dybowski *et al. *[9]. This structural classifier based on the electrostatic potential (RF.ESP) reached an AUC of 0.810 ± 0.008. A subsequent model based on the results of a feature selection described in Materials and Methods yielded an AUC 0.898 ± 0.006 using the 32 most important variables according to the RF importance measure. There are different explanations for the inferiority of this structural classifier: (A) At least some of the drug resistance mechanisms witnessed here are not driven by charge. In comparison, a sequence classifier based on the amino acid net charge descriptor reached an AUC of 0.625 ± 0.000. (B) Inaccurate modeling due to limited sequence length. The influence of neighboring residues (primary or tertiary structure) to the electrostatic potential is neglected. (C) Errors in the template structure. Worthylake *et al. *suggested that the alpha helix formed by the p2 sequence is less stable [44], in contrast to the p2 structure of Morellet *et al. *[35]. The stability of the p2 alpha helix might be overestimated because of a high trifluoroethanol concentration used in the experiments. A wrong template structure might ultimately lead to unnatural side-chain placement. At least the second point also applies to the sequence-based classifiers.

**Figure 3:**
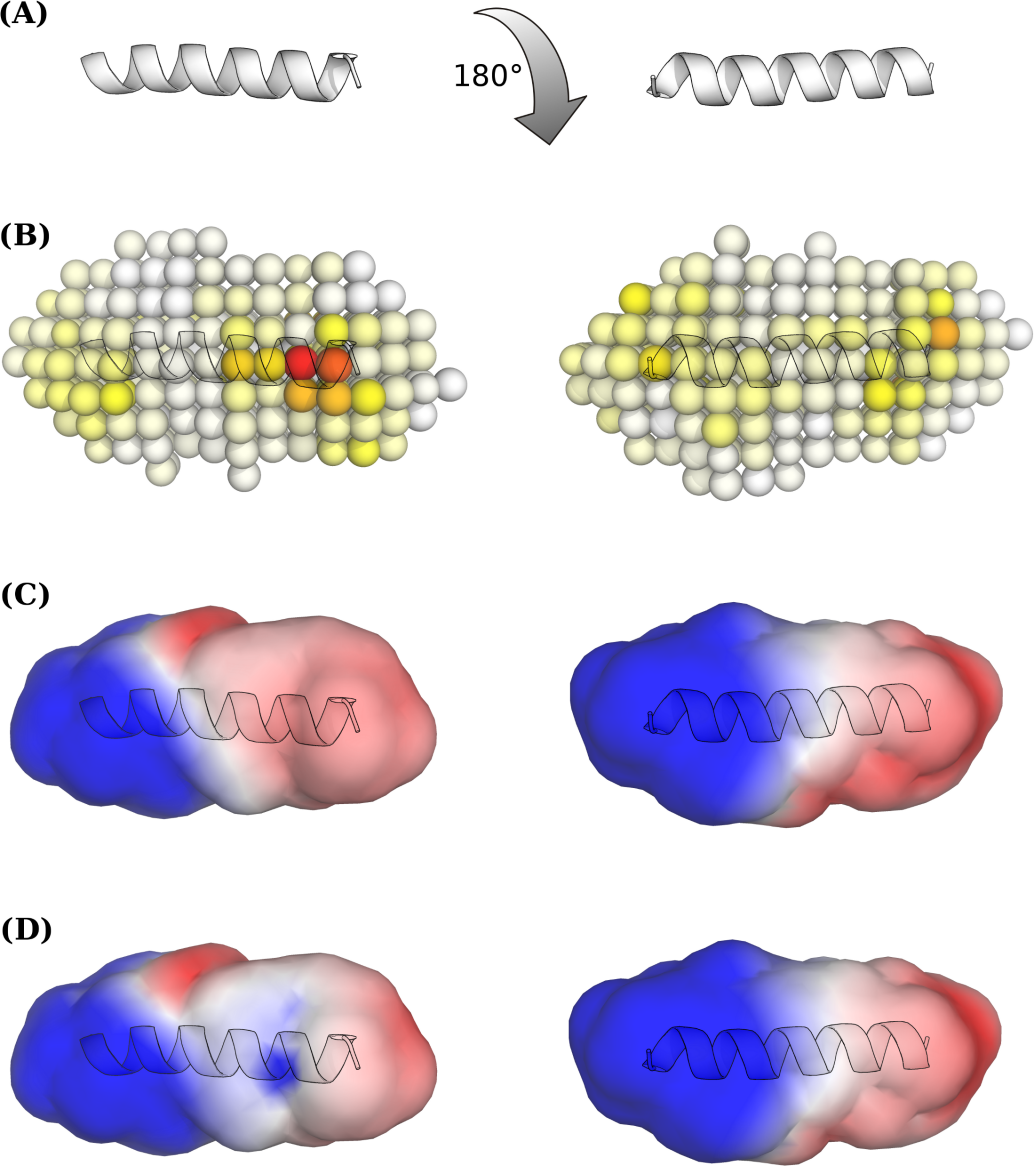
**Electrostatic Hull Importance**. Importance analysis and implications of the electrostatic potential classifier. (A) NMR structure of p2 [35]. (B) Electrostatic hull used as features for model training. Each sphere represents a feature on the hull, colored according to its importance for model performance (white to red). (C) Electrostatic potential at hull averaged over susceptible p2 models. (D) Electrostatic potential at hull averaged over resistant p2 models.

The electrostatics classifier also identified the most important positions mainly in the C-terminal part of the p2 structure, with the potential near positions 373 and 374 being the most important for the classification process (Figure 3). Thus, the ESP classifier is only in partial agreement with the sequence-based classifiers, which identified positions 369-372 as being most important. As already demonstrated by Dybowski *et al. *[9] for co-receptor tropism prediction, the combination of structural and sequence-based classifiers can improve prediction performance significantly. Thus, we tested two scenarios, namely RF.ESP combined with hydropathy scale according to Dybowski *et al. *[9], and a combination of RF.293 with RF.ESP. Combining the ESP- and hydrophobicity-based classifiers produced a slightly improved performance using the product fusion method (AUC = 0.94 ± 0.001). However, a significant increase in AUC was achieved while combining the RF.ESP and RF.293 classifiers applying either the mean or product fusion methods (AUC 0.958 ± 0.001 and 0.956 ± 0.001, respectively).

**Figure 4:**
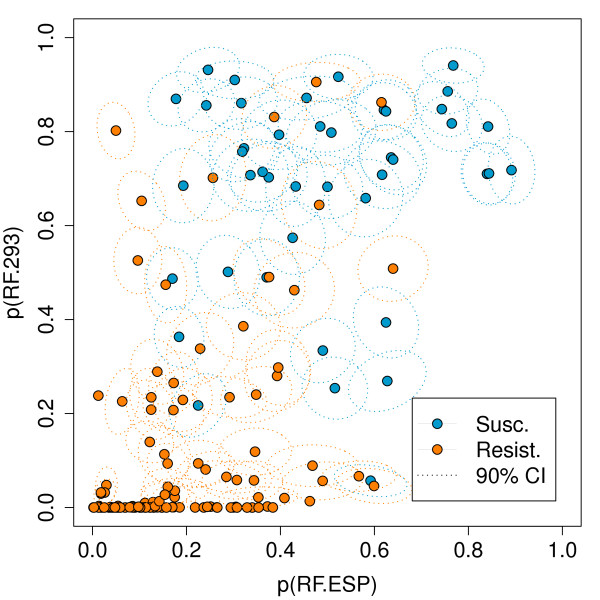
**Class Probabilities**. Vertical and horizontal axis give class probabilities for the p2 sequences from the RF.293 and RF.ESP models, respectively. The dotted lines represent the regions where the resulting votes for a given sequence are to be found with 90% confidence. Confidence regions are shown for sequences where the standard deviation was greater than 0.01 for both models.

**Figure 5:**
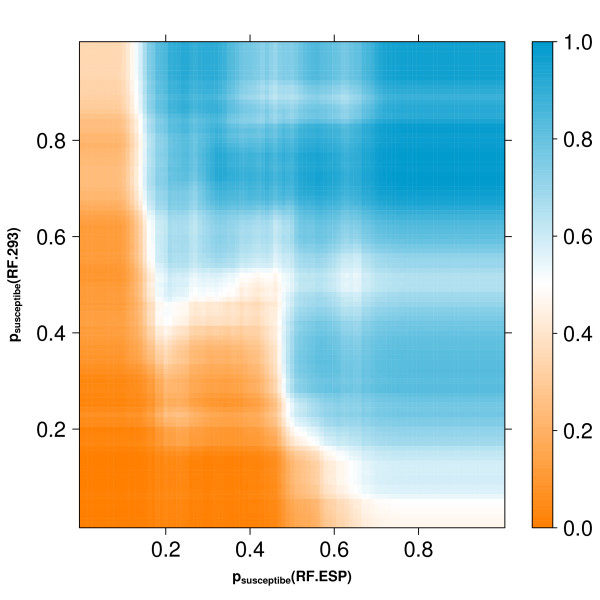
**Prediction Landscape**. Vertical and horizontal axis give all potential class probabilities from the RF.293 and RF.ESP models, respectively. The color marks the output of the second-level learning (stacking).

In Figure 4 the class probabilities according to the two previously described RFs (RF.ESP and RF.293) are plotted for all p2 sequences in the dataset. The figure suggests that the two computational models are in part discordant, as the distribution of both classes, resistant and susceptible, extends into the upper left and lower right corners. This explains the improvement in prediction performance by combining the two RFs. However, stacking leads again to drop in performance (AUC 0.930 ± 0.006). In Figure 5, the potential inputs and the outputs for the second-level learning RF are shown. Obviously, stacking leads to overfitting in some regions, represented by white islands in the prediction landscape in Figure 5, and thus, to a worse generalization ability, reflected in a drop of prediction performance.

## Conclusions

HIV-1 drug resistance is a major obstacle in achieving sustained suppression of viral replication in chronically infected patients. The emergence of drug resistance as well as more and more individualized antiretroviral treatment regimens lead to the need for developing new antiretroviral agents for routine clinical practice. BVM was the first drug of the new class of maturation-inhibitors of HIV-1 entering clinical trials. Baseline BVM resistance of about 30% in treatment-naïve HIV-1 isolates and of about 50% in protease inhibitor resistant HIV-1 isolates [45] hampered the usage of BVM in routine antiretroviral therapy regimens [46]. Nevertheless, new drugs of this new class targeting the p24/p2 junction, e.g. Vivecon (MPC-9055), are already under development and might enlarge the arsenal of antiretroviral drugs in the future. Therefore, highly specific and accurate tools to predict resistance to maturation inhibitors can help to identify patients who might benefit from the usage of these new drugs.

In the current study, we applied several techniques to improve Bevirimat resistance prediction from p2 sequences of HIV-1. Based on our recently published results, we were able to improve resistance prediction with well chosen descriptors and classifier ensembles. It turned out that combining structural and sequence information can lead to improved prediction performance, as already discussed by Dybowski *et al. *[9] for co-receptor usage prediction of HIV-1. Combining well chosen sequence-based descriptors does also lead to better prediction performance with no significant differences compared to the combined structure-sequence classifiers. However, it is not useful to combine plenty of classifiers as it can lead to a drop in prediction performance as demonstrated for the CE1. As already shown in other studies, combining classifiers via stacking seems to be useless to improve prediction performance.

## Competing interests

The authors declare that they have no competing interests.

## Authors' contributions

DH* and JND have jointly carried out computational analyses, interpreted results and drafted the manuscript. MR, SH and MP have contributed software frameworks. JV and DH have revised the manuscript.

All authors read and approved the final manuscript.
